# Determination of the Genome and Primary Transcriptome of Syngas Fermenting *Eubacterium limosum* ATCC 8486

**DOI:** 10.1038/s41598-017-14123-3

**Published:** 2017-10-20

**Authors:** Yoseb Song, Jongoh Shin, Yujin Jeong, Sangrak Jin, Jung-Kul Lee, Dong Rip Kim, Sun Chang Kim, Suhyung Cho, Byung-Kwan Cho

**Affiliations:** 1 Department of Biological Sciences and KI for the BioCentury, Korea Advanced Institute of Science and Technology, Daejeon, 34141 Republic of Korea; 2 Department of Chemical Engineering, Konkuk University, Seoul, 05029 Republic of Korea; 3 Department of Mechanical Engineering, Hanyang University, Seoul, 133-791 Republic of Korea; 4 KAIST Institute for the BioCentury, Korea Advanced Institute of Science and Technology, Daejeon, 34141 Republic of Korea; 5 Intelligent Synthetic Biology Center, Daejeon, 34141 Republic of Korea

## Abstract

Autotrophic conversion of CO_2_ to value-added biochemicals has received considerable attention as a sustainable route to replace fossil fuels. Particularly, anaerobic acetogenic bacteria are naturally capable of reducing CO_2_ or CO to various metabolites. To fully utilize their biosynthetic potential, an understanding of acetogenesis-related genes and their regulatory elements is required. Here, we completed the genome sequence of the syngas fermenting *Eubacterium limosum* ATCC 8486 and determined its transcription start sites (TSS). We constructed a 4.4 Mb long circular genome with a GC content of 47.2% and 4,090 protein encoding genes. To understand the transcriptional and translational regulation, the primary transcriptome was augmented, identifying 1,458 TSSs containing a high pyrimidine (T/C) and purine nucleotide (A/G) content at the −1 and +1 position, respectively, along with 1,253 5′-untranslated regions, and principal promoter elements such as −10 (TATAAT) and −35 (TTGACA), and Shine-Dalgarno motifs (GGAGR). Further analysis revealed 93 non-coding RNAs, including one for potential transcriptional regulation of the hydrogenase complex via interaction with molybdenum or tungsten cofactors, which in turn controls formate dehydrogenase activity of the initial step of Wood-Ljungdahl pathway. Our results provide comprehensive genomic information for strain engineering to enhance the syngas fermenting capacity of acetogenic bacteria.

## Introduction

Demands for renewable and sustainable alternatives for fossil resources have rapidly increased along with concerns about environmental issues. A number of methods have been suggested for replacing fossil resources, but most of them demonstrate limitations in terms of lack of environmental, social, and economic sustainability^1^. An alternative approach is to utilize the metabolism of acetogenic bacteria to convert CO_2_ into biomass and commodity chemicals; this is considered a promising approach to reduce and potentially replace fossil resources^2^. Representing 23 different genera, acetogenic bacteria robustly synthesize acetate and biomass from chemolithoautotrophic substrates^3^. In particular, *Eubacterium limosum* ATCC 8486, which is a gram-positive obligate anaerobic bacterium isolated from the rumen fluid of sheep and a commercially available type strain, is reported to convert syngas, such as CO, CO_2_, and H_2_, to butyrate, caproate, and acetate via the reductive acetyl-CoA pathway, also called the Wood-Ljungdahl pathway (WLP)^4–6^. In addition, syngas fermentation using this strain has advantages over the traditional thermochemical process of the Fischer-Tropsch synthesis, including higher catalyst specificity, lower operational temperature, higher tolerance of impurities, and lower energy costs^7–9^. Furthermore, the strain is capable of biotransformation of biochanin A, formononetin, and glycitein to multiple estrogenic metabolites with anticancer, antioxidant, anti-inflammatory, and enzyme inhibitory properties^10^.

Despite several reports on the genomic information and understanding of biochemical mechanism of WLP, studies on acetogenic bacteria have mostly focused on discovery of gene contents. Along with gene content information, DNA sequences such as transcription start sites (TSSs), principal promoter elements, such as −10 and −35 motifs, 5′-untranslated regions (UTRs), and Shine-Dalgarno (SD) sequences are *cis*-encoded determinants for transcriptional and translational regulation embedded in the genome^11^. The precise locations of TSSs determined by experimental methods provide the sequence and structure of the 5′ end of mRNA for investigating transcriptional regulation, mRNA stability, and translational efficiency. To understand the regulation of syngas fermentation by acetogenic bacteria, it is important to comparatively analyse the genomic features among acetogenic bacteria and determine the genome embedded non-coding regulatory elements, such as promoters, 5′UTRs, and non-coding RNAs (ncRNA), which play key roles in transcriptional and post-transcriptional regulation.

In this study, we analysed the complete genomic sequence and determined the genome-wide TSSs of *E*. *limosum* ATCC 8486. To this end, we integrated a long-read sequencing and short-read sequencing platform to obtain an accurate genomic sequence, and then identified the metabolic pathways responsible for syngas fermentation. In addition, we determined, for the first time in acetogenic bacteria, the primary transcriptome to unravel the transcriptional landscape using differential RNA sequencing (dRNA-Seq). Based on the integrated analysis with the complete genomic sequence, we suggest that the regulatory features such as principal promoter elements and 5′UTRs orchestrate the WLP and energy conservation system of *E*. *limosum*.

## Results

### Completion of genome sequence using single molecule real-time (SMRT) sequencing

To complete the genome sequence of *E*. *limosum* ATCC 8486, we exploited SMRT sequencing and a hierarchical genome assembly process pipeline^12^. In total, 8,986 reads with 994,287,089 bases were obtained, which were assembled into one circular contig comprising 4,423,093 bases. Compared to the previously assembled draft genome that consisted of 31 contigs with 4,370,113 bases, the newly completed genome sequence is 52,980 bases longer, but with similar GC content (Supplementary Table 1).

Despite completing the genome sequence, SMRT sequencing often presents random errors, which may result in incorrect sequences that might be part of the assembled genome^13^. To correct potential sequence errors in the assembled contig, we mapped 38,752,997 short reads (150 bp in average length) obtained from the Illumina sequencing onto the assembled circular contig, resulting in 121 nucleotide conflicts with 106 deletions and 15 mismatches (Supplementary Table 2)^14^. The conflict sites were further compared with the RNA-Seq and Sanger sequencing results, indicating that the Illumina short read sequences were correct. In the corrected genome sequence, the origin of replication was determined at a genomic position of 689,833, with the terminus replication position at 3,794,077 (Supplementary Fig. 1). Along with a GC content of 47.2%, the annotation resulted in 4,281 genes comprising 4,090 protein-encoding genes with 64.5% putative functions, 16 ribosomal RNAs (rRNA) and 60 transfer RNAs (tRNA) (Table 1 and Supplementary Table 3).

**Table 1:** General features of the *E*. *limosum* genome.

Feature	Number (% of Total)
Genome size (base pairs)	4,422,837 bp
G + C content	2,087,152 bp (47.2%)
Genes	4,281
Predicted protein encoding sequences	4,090 (95.5%)
Predicted sequences encoding RNA genes	191 (44.6%)
Coding density	3,918,026 bp (88.6%)
rRNA	16 (0.4%)
tRNA	60 (1.4%)
Genes with function prediction	2,763 (64.5%)
Genes assigned to COGs	3,793 (88.6%)
Genes with Pfam domains	2,820 (65.9%)
Genes with signal peptides	276 (6.4%)

### Comparative genome analysis using pan-genome analysis

**Figure 1:**
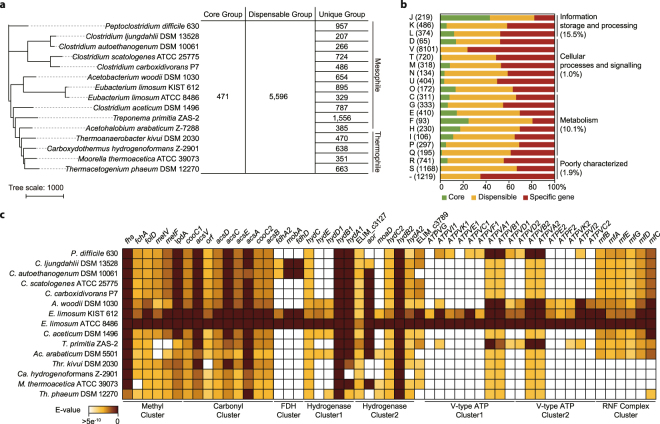
Pan-genome analysis of *Eubacterium limosum* ATCC 8486. (**a**) A pan-genome tree was constructed using 15 acetogenic bacterial genomes with several core, dispensable, and unique gene groups. (**b**) Distribution of core (green), dispensable (yellow), and specific genes (red) based on Clusters of Orthologous Groups category. (**c**) Comparison of proteins associated with the Wood-Ljungdahl pathway and energy conservation system between *E*. *limosum* ATCC 8486 and 15 acetogenic bacteria was performed using BLAST. Genes with E-value lower than 5^−10^ are coloured as indicated by the colour key. Abbreviation: A, *Acetobacterium*; Ac, *Acetohalobium*; Ca, *Carboxydothermus*; C, *Clostridium*; E, *Eubacterium*; M, *Moorella*; P, *Peptoclostridium*; T, *Treponema*; Th, *Thermacetogenium*; The, *Thermacetogenium*.

To explore the common and unique genetic basis of *E*. *limosum* ATCC 8486 compared to other acetogenic bacteria^3,15^, we next analysed the completed genomes using pan-genome analysis. The genes were categorized into core, dispensable, and specific genes present in all strains, two or more strains, and unique strains, respectively, which reveals the conserved and novel genes in the newly sequenced genomes^16^. In this manner, pan-genome analysis provides genetic background information to understand the physiological diversity in acetogenic bacteria^17,18^. The distribution of gene families in the completed genome sequences of acetogenic bacteria (15 genomes in total) included 54,268 genes with 15,436 orthologous groups, composed of 471 core gene groups with 13,513 genes, 5,596 dispensable gene groups with 31,387 genes, and 9,368 unique genes (Fig. 1a, Supplementary Table 4, and Supplementary Table 5).

Based on the pan-genome tree analysis, *E*. *limosum* ATCC 8486 is closely related to *E*. *limosum* KIST 612 and *Acetobacterium woodii*, in agreement with the previous 16 S rRNA sequence-based analysis^18,19^. However, their different metabolic properties illustrate the physiological and phylogenetic diversity of acetogenic bacteria (Fig. 1a). Functional classification of orthologous groups ascertained that genes responsible for information storage and processing, especially translation, are the most conserved, including WLP-associated genes. In contrast, genes encoding cellular processes and signalling showed low conservation, especially with defence mechanisms, indicating a genetic basis for the physiological diversity of acetogenic bacteria (Fig. 1b). *E*. *limosum* ATCC 8486 represents 1,004 core genes, 2,757 dispensable genes, and 329 unique genes. The core genes were well annotated (95.3%); however, 49.2% unique genes were identified with hypothetical functions (Supplementary Fig. 2).

The WLP-associated genes were detected in the core gene groups (Fig. 1c and Supplementary Table 5). The WLP is initiated by reduction of CO_2_ to formate catalysed by formate dehydrogenase (FDH). An FDH-encoding gene (ELIM_c2470) was identified in the core gene group, which is clustered with genes encoding the molybdopterin-guanine dinucleotide biosynthesis protein (ELIM_c2471) and an FDH-accessory protein (ELIM_c2472)^3,15^. Similar FDH cluster organization, which contains Fdh, Mob, and FdhD, in that order, was observed in *Clostridium ljungdahlii* (CLJU_c06990-CLJU_c07020), *C. autoethanogenum* (CAETHG_2790-CAETHG_2793), and *E*. *limosum* KIST 612 (ELI_0994-ELI0997). In contrast to the direct association of FDH with an additional hydrogenase subunit for the generation of reducing equivalents from H_2_ for CO_2_ reduction in *A*. *woodii*, the gene clusters encoding hydrogenases in *E*. *limosum* ATCC 8486 were located in at different genomic regions. Despite the different FDH complex identified, the putative electron-bifurcating hydrogenase (ELIM_c2347-ELIM_c2351) was similar to the one in *A*. *woodii* (Awo_c27010-Awo_c26790). In addition, two gene clusters encoding putative hydrogenases were predicted (ELIM_c3789 and ELIM_c3129-ELIM_c3132). Interestingly, most genes (six out of eight genes) in the putative hydrogenase gene clusters were included in the core gene groups (Fig. 1c and Supplementary Table 5).

To reduce the formyl-group to a methyl-group, *E*. *limosum* ATCC 8486 contains a gene cluster for the methyl branch, comprising six genes (ELIM_c0957-ELIM_c0962) as core gene groups, encoding the formyl-THS synthetase (*fhs1*), methenyl-THF cyclohydrolase (*fchA*), methylene-THF dehydrogenase (*folD*), methylene-THF reductase (*metV* and *metF*), and dihydrolipoyl dehydrogenase (*lpdA*). Despite a similar gene arrangement of methyl branch coding genes, the only difference with the gene cluster of the methyl branch in *A*. *woodii* is the absence of an additional *rnfC2* encoding electron transport complex subunit (Fig. 1c)^20^. In contrast to the genes encoding methyl and carbonyl branches that are organized in one cluster in genus *Clostridium* ^18,21^, the gene cluster (ELIM_c1647-ELIM_c1655) for the carbonyl branch, required for acetyl-CoA formation from methyl-THF and a second CO_2,_ was located at genomic regions different from the gene cluster for the methyl branch (Supplementary Fig. 1). The CO dehydrogenase/acetyl-CoA synthase complex is encoded by *acsA* (ELIM_c1653) and *acsB* (ELIM_c1655), together with the genes encoding the corrinoid/iron sulphur protein (*acsCD*), the methyltransferase (*acsE*), the CODH nickel-insertion accessory proteins (*cooC1* and *cooC2*), the corrinoid activation and regeneration protein (*acsV*), and a hypothetical protein (Fig. 1c).

Energy metabolism in acetogenic bacteria is largely classified into two membrane-bound electron transport systems; a proton dependent cytochrome-containing transmembrane and a proton/sodium ion-translocating ferredoxin:NAD^+^ oxidoreductase (Rnf)^22–24^. In the *E*. *limosum* genome, similar to the electron transfer system found in *A*. *woodii*, all the genes encoding the Rnf complex were identified (ELIM_c3879-ELIM_c3884) and the cytochrome coding genes were not, which were classified as dispensable gene groups (Fig. 1c and Supplementary Table 5). Recently, the Na^+^ dependent activity of the membrane-bound Rnf complex was successfully measured by ferredoxin-dependent NAD^+^ reduction in *E*. *limosum* KIST 612^25^.

For subsequent ATP synthesis using the established ion gradient, *E*. *limosum* ATCC 8486 potentially utilizes Na^+^ for translocating ATP synthase, which contains an Na^+^ binding motif in the *c* subunit (Supplementary Fig. 3). In contrast to most of the acetogenic bacteria exploiting the F-type ATP synthase complex, only A/V-type ATP synthase complexes were identified that are similar to the complex found in *A*. *woodii* ^20^. Taken together, the pan-genome analysis demonstrates that *E*. *limosum* ATCC 8486 exhibits an energy conservation system phylogenetically similar to that of *A*. *woodii* and an FDH complex similar to that of *C*. *ljungdahlii*.

### Determination of the primary transcriptome landscape using dRNA-Seq

**Figure 2:**
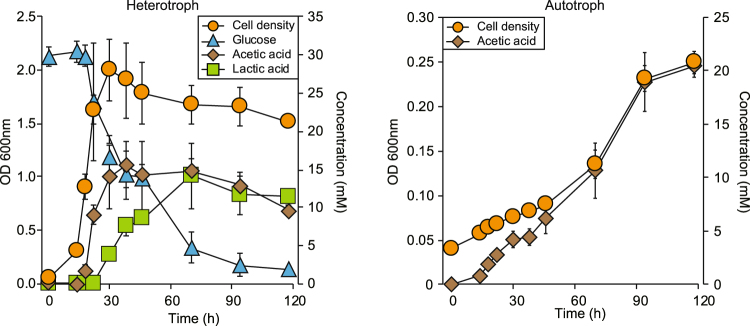
Growth of *Eubacterium limosum* ATCC 8486 under heterotrophic and autotrophic conditions. (**a**) Cell density (orange circle), substrate consumption of glucose (blue triangle), and production of acetic acid (brown rhombus) and lactic acid (green rectangle) in the heterotrophic growth of *E*. *limosum*. (**b**) Cell density of *E*. *limosum* (orange circle) and production of acetic acid (brown rhombus) in the autotrophic growth.

With the completed genome sequence and newly annotated genes, we profiled the sites of transcription initiation using dRNA-Seq. TSS maps of bacterial genomes provide a basis to comprehend the interconnected regulatory components of bacterial genomes such as promoter elements, enabling us to analyse bacterial operons and transcription units^26–28^. In addition, dRNA-Seq has been applied to stipulate the presence of regulatory ncRNAs^26–28^. To this end, total RNA samples were obtained from cells grown at mid-exponential phase under heterotrophic (5 g L^−1^ glucose) and autotrophic (H_2_/CO_2_ (80:20) at a pressure of 200 kPa) conditions with generation times of 0.96 h and 9.38 h, respectively. Under heterotrophic conditions, glucose consumption was completed to reach an optical density of 1.63 ± 0.11 at wavelength 600 nm (OD_600nm_) and to produce acetate (11.62 ± 1.33 mM) as well as lactate (12.00 ± 3.43 mM), with a maximal optical density (OD_600nm_) of 2.00 ± 0.28 after 30 h (Fig. 2a). Under autotrophic conditions, a maximal optical density (OD_600nm_) of 0.26 ± 0.01 was reached after 118 h and only acetate (20.46 ± 1.02 mM) was detected as a metabolic end product at higher levels than those produced under the heterotrophic conditions (Fig. 2b).

**Figure 3:**
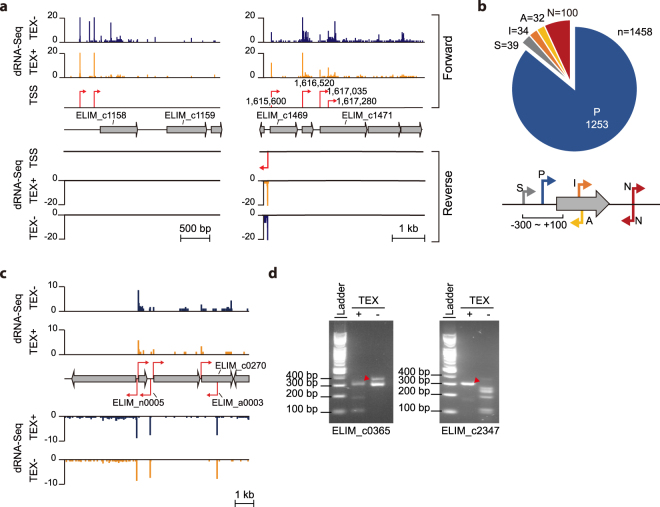
Transcriptional architecture of the *E*. *limosum* genome. (**a**) Example of TSS sites in *E*. *limosum* genome under heterotrophic and autotrophic conditions. Two libraries of terminator-5′-phosphate-dependent exonuclease treated (TEX+) and only RNA 5′-polyphosphatase treated (TEX-) were constructed. (**b**) TSSs located in the region between 300 bp upstream and 150 bp downstream of the corresponding start codon of each ORF were determined. Of the obtained sites, TSSs were classified as primary (**P**) or secondary (**S**). TSSs identified within the ORF, on the opposite strand, and all the others were classified as internal (**I**), antisense (**A**), and intergenic (**N**), respectively. In total 1,458 TSSs were identified. (**c**) ncRNA in the antisense of ELIM_c0270 (ELIM_a0003), which is located on the reverse strand of ELIM_c0270, and intergenic ncRNA (ELIM_n0005), which is located downstream of ELIM_c0194, were identified as ncRNAs. (**d**) TSS confirmation of glycerol-3-phosphate dehydrogenase (ELIM_c0365) and NADH dehydrogenase (ELIM_c2347) associated genes using rapid amplification of 5′ cDNA ends. The red triangle indicates the target confirmation length. The full-length gels are presented in Supplementary Fig. 6.

The isolated total RNA samples were treated with terminator exonuclease (TEX) (see Material and Methods for culture and sampling conditions). TEX treatment selectively enriches the 5′ triphosphorylated RNAs (i.e., the primary transcriptome) by degrading the processed or degraded transcripts^27,28^. After subsequent treatment with RNA 5′-polyphosphatase (TAP) for adapter ligation, we prepared two cDNA sequencing libraries, which were generated from TEX-treated (TEX+) and non-treated (TEX−) RNA, respectively. Approximately, 1.4 million sequence reads were uniquely mapped to the genome sequence with an average read length of 90.6 bp corresponding to a 33-fold genomic coverage **(**Supplementary Table 6 **)**. This was followed by data integration of the TEX+ and TEX− libraries with the criteria that selectively identified the 5′ ends of primary transcripts (see the Methods for TSS mapping criteria). As illustrated in Fig. 3a, the 5′ ends of primary transcripts were selectively determined, providing a total of 1,458 TSSs in the genome (Supplementary Table 7).

The obtained TSSs were further classified by their positions relative to corresponding coding sequences, yielding 1,253 primary TSSs with 30.6% of the currently annotated genes (note that monocistronic and operonic structures have not been considered) (Fig. 3b). Transcription of 39 genes was initiated by multiple TSSs and 34 internal TSSs were identified within 34 genes. In addition, 100 TSSs were found in intergenic regions without an association with the currently annotated genes and 32 TSSs were identified from the antisense strands of 32 genes, suggesting the presence of regulatory RNAs (Supplementary Table 7). For example, putative ncRNAs (ELIM_n0005 and ELIM_a0003) were found on the antisense strand of intergenic regions between ELIM_c0268 and ELIM_c0269, and ELIM_c0270, respectively (Fig. 3c). To validate the identified TSSs, they were independently tested by rapid amplification of 5′ cDNA ends (5′-RACE) (Fig. 3d, Supplementary Fig. 4, and Supplementary Fig. 5)^29^. Taken together, we have identified an average of one TSS for every three currently annotated genes, which is further used to identify the promoter sequences within the upstream regions of individual genes.

### Identification of regulatory sequences within the upstream regions of genes

**Figure 4:**
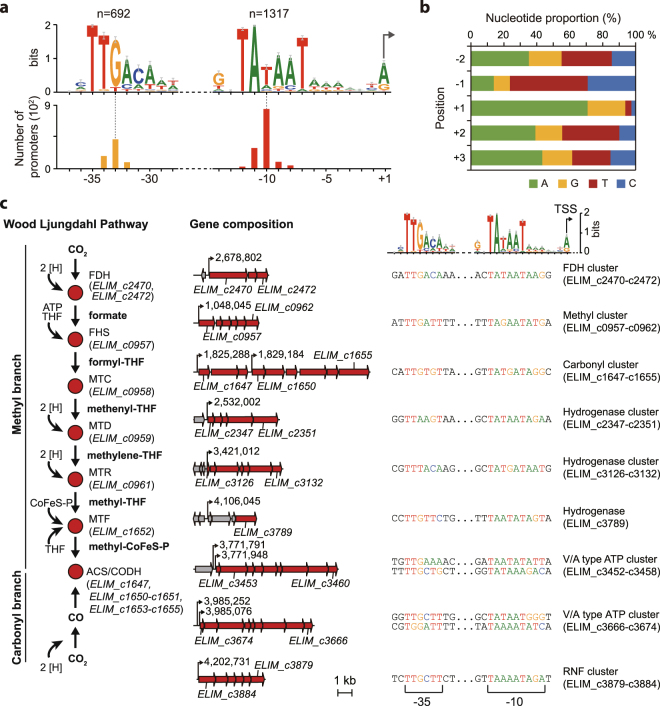
Genome-scale analysis of upstream sequences. (**a**) The −10 motif (5′-TATAAT-3′) and −35 motif (5′-TTGACA-3′) were identified relative to the TSS position (+1). The conserved motif is identical to the housekeeping sigma factor (*rpoD*) binding site of *Bacillus subtilis* promoters. (**b**) TSS nucleotide ratio at +1 and 2 bp upstream and downstream of TSSs. (**c**) Promoter sequences of acetogenesis associated clusters. The −35 motif (5′-TTGACA) and −10 motif (5′-TATAAT) were identified as promoter sequences of the methyl branch of Wood-Ljungdahl pathway, carbonyl branch of WLP, formate dehydrogenase (FDH), hydrogenase, ATP synthase complex, and Rnf complex clusters, relative to the TSS position (+1).

The bacterial RNA polymerase (RNAP) is sufficient to synthesize RNA transcripts in bacteria: however, the formation of RNAP holoenzyme with sigma factor (σ factor) is essential to recognize the specific promoter sequence^30,31^. Thus, determination of σ factor-specific promoter sequences is important to understand the transcription initiation and transcriptional regulation in response to environmental conditions. Genome annotation accounts for seven σ factors (*rpoD* (ELIM_c3331), *rpoN* (ELIM_c0109), *sigH* (ELIM_c1247), *sigB* (ELIM_c2321), *sigR* (ELIM_c3151), *sigE* (ELIM_c3281), and *sigV* (ELIM_c3405)). To determine the promoter elements in the genome, the consensus sequence motifs were searched from 50 bp upstream of the identified TSSs using MEME software^32^. The consensus sequence motifs were composed of three boxes (i.e., −10, −14, and −35 boxes) often found in the promoter regions of *B*. *subtilis* ^33^. The −10 motif (TATAAT) and −35 motif (TTGACA) for the housekeeping σ factor were conserved in 90.3% (1,317 out of 1,458; *P* < 0.05) and 47.5% (692 out of 1,458; *P* < 0.05) of the identified TSSs, respectively, with an internal length between 16 and 18 bp (Fig. 4a). This indicates that the identified promoter motifs are regulatory sequences for the housekeeping σ factor (RpoD), identical to the RpoD motif in *E*. *coli* and *B*. *subtilis* ^34,35^. At the −14 site, high content of T/G sequence was found, which is like a sequence motif located in the promoter of *B*. *subtilis* ^34^. The −1 site was rich in pyrimidine nucleotide content (T/C) with 86.3% pyrimidines whereas, the +1 site was rich in purine nucleotide content (A/G) with 93.8% purines, consistent with the common observation from promoters in archaea, bacteria, and mammalian cells (Fig. 4b). With this pyrimidine-purine dinucleotide preference, consensus sequence motifs for the housekeeping σ factor were found in the promoter regions of gene clusters encoding FDH, hydrogenases, carbonyl and methyl branches, Rnf, and ATP synthase (Fig. 4c). Based on the identified primary TSS, the methyl branch cluster and the carbonyl branch cluster are considered as one operon and two operons, respectively. The first operon of the carbonyl cluster is composed of the CODH accessory protein (*cooC1*), corrinoid activation/regeneration protein (*acsV*), and a hypothetical protein (ELIM_c1649). Interestingly, the first two genes, *cooC1* and *acsV*, in the first cluster of *E*. *limosum*, are located in both flanks of the carbonyl branch gene (*acsD*, *acsC*, *acsC*, *acsE*, *acsB*, and *gcvH*) of acetogenic *Clostridium* species, indicating that transcriptional regulation of the WLP genes may vary throughout acetogenic bacteria.

**Figure 5:**
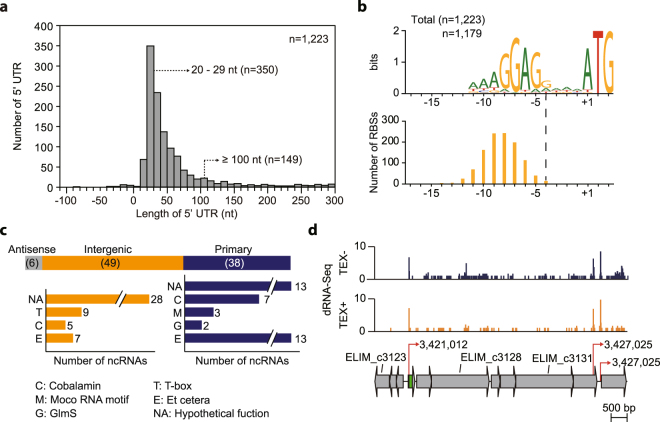
Genome-scale analysis of 5′UTR and functional prediction of regulatory ncRNAs. (**a**) Distribution of 1,223 5′UTRs of *E*. *limosum*, with a major peak at length 20–29 nt. (**b**) The conserved ribosome binding site (RBS) motif (GGAGR) for protein coding genes in the 5′UTRs is longer than 8 nt. The bottom panel shows conservation of each motif of RBS. (**c**) Classification of ncRNAs using antisense, intergenic, and primary TSSs, according to their locations. The functions of the classified RNAs were predicted using the Rfam database. (**d**) Moco-RNA motif containing ncRNA, which was identified by dRNA-Seq, was located upstream of the hydrogenase complex at genome position 3,421,126, which was confirmed via the Rfam database.

The 5′UTR of mRNA plays an important role in transcriptional and translational regulation. Using the primary TSSs, 5′UTR length was calculated and we found that 28.6% were in the interval length of 20-29 nt and 87.8% were found to be shorter than 100 nt (Fig. 5a). In addition, 149 TSSs with 5′UTR lengths longer than 100 nt were found, which potentially form RNA structures for post-transcriptional regulation^36^. Interestingly, the methyl cluster, first carbonyl cluster, hydrogenase cluster (ELIM_c2347-ELIM_c2351), both ATP synthases, and Rnf complex contain relatively longer 5′UTRs (41-64 nt). We searched for ribosome binding sites (RBS) in 5′UTRs from −17 to +3 nt of the corresponding start codons. The alignment yielded a highly conserved motif (*P* < 0.05), identifying the GGAGR sequence similar to motifs in many different bacterial genomes (Fig. 5b)^37,38^. The spacer length from the starting codon to RBS was between 5 nt and 10 nt (91.0%), which was previously reported to be an optimal space for translation in bacterial cells^39^. The 5′UTRs responsible for WLP and FDH gene clusters are composed of RBS sequences of AGGAGG, a spacer 7-9 nt long, and ATG as the starting codon (Supplementary Table 8). Similarly, all clusters of the Rnf complex, ATP synthase complex, and hydrogenase complex (ELIM_c2347) have spacers of length between 6 and 7 nt with an RBS sequence of AGGAGG.

### Functional prediction of ncRNAs in acetogenesis related gene clusters

To understand the functional roles, ncRNAs were predicted using the Rfam database^40^, resulting in a total of 107 regulatory RNAs (Supplementary Table 9). Among the predicted RNAs, 93 were evidenced by the presence of TSSs, of which 38 TSSs were classified as primary, 49 were intergenic, and six were antisense. Of the 38 ncRNAs classified by primary TSSs, nine were identified as T-box elements, reported to be located upstream of the amino acid biosynthesis encoding genes and responsible for translational regulation of downstream genes; five were characterised as the cobalamin riboswitches, which are reported to be located in the 5′UTR of the vitamin B12 (cobalamin) encoding gene to regulate gene expression and play an important role in acetogenic bacteria growth under autotrophic conditions^41–43^, and 28 had unknown functions (Fig. 5c). Among the 49 RNAs determined by intergenic TSSs, seven were cobalamin riboswitches, three were molybdenum or tungsten sensing motifs (Moco-RNA motif), and two were cyclic di-GMP-I riboswitches, which regulate virulence, motility, and biofilm formation in various bacteria (Fig. 5c)^44^. All the RNAs confirmed by antisense TSSs were annotated as unknown functions.

One RNA, confirmed by primary TSS (ELIM_c2254), was positioned upstream of the cobalamin biosynthesis encoding genes (ELIM_c2254-ELIM_c2264) responsible for synthesizing cobyrinic acid a,c-diamide from precorin-2. The other cobalamin RNA, confirmed by intergenic TSS (ELIM_n0060), was located upstream of the other cobalamin encoding genes (ELIM_c2270-ELIM_c2283) that convert glutamate to precorrin-2 and threonine to adenosyl cobinamide phosphate. Although the cobalamin encoding operons were nonadjacent, they were proximal and separated by 6,009 bp, containing their own cobalamin regulating RNAs, which suggests that the cell closely orchestrates the expression of the pathway^41^. Along with regulation of the cobalamin encoding genes, T-box elements were found upstream of amino acid associated genes, such as isoleucyl-tRNA synthetase (ELIM_c0212), putative ABC transporter (ELIM_c0563-ELIM_c0565), methionyl-tRNA synthetase (ELIM_c0596), 2-isopropylmalate synthase (ELIM_c1326), alanyl-tRNA synthetase (ELIM_c1518), threonyl-tRNA synthetase (ELIM_c3489), tryptophan synthase (ELIM_c3008-ELIM_c3014), and precursors for branched-chain amino acids (ELIM_c3959-ELIM_c3965). In addition, Moco-RNA motif was located upstream of the hydrogenase complex cluster (ELIM_c3126-ELIM_c3132), which is predicted to reduce FDH that is responsible for the initial step of WLP by converting CO_2_ into formate (Fig. 5d). Moco-RNA motif was reported to regulate downstream gene expression by sensing the concentration of molybdenum or tungsten available in the cell, indicating that the gene expression of the hydrogenase complex might be controlled by these metals^45^. Interestingly, molybdenum and tungsten were reported to regulate gene expression of FDH as well as enhance its catalytic efficiency via metal incorporation^46^. Based on the circumstances, it is hypothesized that the presence of the motif potentially regulates the hydrogenase complex in order to balance FDH to improve efficiency of the initial step of WLP.

## Discussion

Acetogenic bacteria inhabit diverse environments and hence culture conditions vary, indicating that the regulatory elements embedded in their genomes may differ across strains. Among them, the genome sequence of *E*. *limosum* ATCC 8486 was completed, and was found phylogenetically close to *A*. *woodii*. Genes encoding the methyl and carbonyl branches of the WLP were located in two different genomic regions, whereas a single gene cluster encodes both methyl and carbonyl branch in other acetogenic *Clostridia* ^21^. The gene arrangement of clusters was almost identical to that of gene clusters in *A*. *woodii*, except an additional copy of a hypothetical gene in the carbonyl branch and an absence of the *rnfC* gene in the methyl branch. In addition, the energy conservation system was similar to the system in *A*. *woodii*, containing two gene clusters encoding ATP synthase complexes and utilizing the Rnf complex as the electron transfer system for reduction of the electron carrier^20^. It is unclear which ion in the Rnf complex translocates but based on the presence of an Na^+^ binding motif, the complex probably generates sodium chemiosmosis. Despite being phylogenetically related to *A*. *woodii*, based on its FDH and hydrogenase composition, *E*. *limosum* is incapable of hydrogen-dependent CO_2_ reduction; it instead accepts electrons from ferredoxin similar to *C*. *ljungdahlii*. Phylogenetically related acetogenic bacteria demonstrate different acetogenesis characteristics, indicating that the WLP and energy conservation system compartments should be considered as modules and acetogenic bacteria probably adopted parts that fit in their own environments.

The primary transcriptome landscape provides an understanding of the transcriptional and translational regulation of acetogenesis in autotrophic conditions. Although the complexity of TSSs varies in organisms, less than 10% of the total TSSs in *E*. *limosum* show multiple usage in an ORF, whereas bacteria inhabiting a wide range of environments are reported to utilize significantly more TSSs, which indicates less complexity of transcriptional regulation in the strain compared to other generalist bacteria^27,47^. However, a wide range of ncRNAs were identified in the antisense and non-coding regions, which are involved in post-transcriptional regulation^48^. Interestingly, one of the ncRNAs identified upstream of the hydrogenase complex (ELIM_c3126 – ELIM_c3132) was predicted to play a key role in reducing the electron carriers in autotrophic conditions. The function of Moco-RNA motif that regulates transcriptional expression of the downstream gene depends on the concentration of molybdenum or tungsten cofactor, which is involved in increasing the efficiency of FDH^46,49^. Thus, *E*. *limosum* may balance the expression of FDH and the hydrogenase complex by using the availability of cofactors to minimize energy wastage during transcription in the autotrophic conditions.

5′UTRs are reported to play an important role by forming structures during post-transcriptional regulation^50,51^. Interestingly, the lengths of 5′UTRs that are responsible for regulating WLP were longer than the median 5′UTR length (36 nt), suggesting that transcriptional expression might be regulated by RNA structure in the 5′UTR^52^. This questions the post-transcriptional regulation of WLP and energy conservation system in autotrophic conditions, which must be approached with the integration of transcriptome and translatome analysis^53^. In this study, the integration of genome and primary transcriptome contributes to an insight on the genomic features between acetogenic bacteria and *E*. *limosum* metabolism. Furthermore, determination of 1,458 TSSs provides a list of promoter sequences, RBS, 5′UTRs, and ncRNAs that predict the transcriptional regulatory system, along with potential post-transcriptional regulation. Together with the results, this information will serve as an important resource for understanding syngas fermentation and strain designing for obtaining better productivity.

## Methods

### Bacterial strains and growth conditions

*E*. *limosum* ATCC 8486 was obtained from the Leibniz Institute DSMZ-German Collection of Microorganisms and Cell Cultures (DSMZ, Germany) and cultivated anaerobically at 37 °C in DSM 135 media supplemented with 5 g L^−1^ glucose or H_2_/CO_2_ (80:20) at a pressure of 200 kPa for heterotrophic and autotrophic growth conditions, respectively. After harvesting by anaerobic centrifugation, the collected cells were washed with basal DSM 135 media and inoculated in 100 mL of fresh medium.

### Genome sequencing

The sequencing library with an average size of 20 kb was sequenced using the PacBio system with Polymerase version P6, and C4 chemistry. Using SMRT Analysis v2.3.0, raw reads with a read quality lower than 0.75 were filtered out, resulting in 1,101,693,824 bases and 105,034 reads with an average length of 10,488 bp. *De novo* assembly of the filtered reads was performed using HGAP.2 SMRT Analysis v2.3.0, giving rise to one contig in a circular form. The highly accurate sequence reads obtained from previous Illumina genome sequencing were then used to correct the random errors in the assembled contig^13^. In total, 5,826,333,852 bases were mapped onto the *de novo* assembled genome with default parameters (mismatch cost = 2, insertion cost = 3, deletion cost = 3, length fraction = 0.5, and similarity fraction = 0.8). Using the Extract Consensus Sequences program in CLC Genome Workbench, conflict sites (mismatches, insertions, and deletions) were detected in the assembled contig. The conflict sites were further confirmed using Sanger Sequencing.

### Gene prediction and annotation

Using the error corrected genome, the origin and terminus of replication were determined using GenSkew (http://genskew.csb.univie.ac.at). tRNA and rRNA were predicted using tRNAscan-SE1.31^54^ and RNAmmer 1.2^55^, respectively. Genome prediction and annotation were performed using Prodigal^56^ and the Swiss-Prot (from Uniprot) database, respectively. The other unknown predicted genes were annotated using the non-redundant protein database of GenBank. Subsequently, the amino acid sequences of annotated genes were aligned against the protein database to identify Gene Ontology (GO), KO ID, and Clusters of Orthologous Groups (COG)^57,58^.

### Pan-genome analysis

In total, 15 complete acetogen genomes, including 14 acetogen genomes downloaded from the National Centre for Biotechnology Information database (ftp://ftp.ncbi.nih.gov/genomes/genbank/bacteria) and the completed genome of *E*. *limosum* ATCC 8486, were analysed using the Pan-Genomes Analysis Pipeline (PGAP-1.12); the core genome, dispensable genomes, and unique genomes were then identified^16^. The translated protein sequences were compared using BLASTP with a minimum score of 50 and *E*-value of 10^−10^. The results were then clustered using the Markov cluster algorithm^59^. The phylogenetic tree of the acetogens was constructed based on the pan-genome pool using neighbour-joining method.

### Differential RNA-Seq (dRNA-Seq) library preparation

The rRNA-depleted RNA was split into three samples for constructing three different libraries (TEX+, TEX−, and TAP−). For preparing the TEX+ library, Terminator^TM^ 5′-Phosphate-Dependent Exonuclease (Epicentre, Madison, USA) was treated with 2 µL of 10× Terminator^TM^ Reaction Buffer A (Epicentre, Madison, USA) and 20 U of SUPERase In RNase Inhibitor (Thermo Scientific, Waltham, USA). For the preparation of TEX− and TAP− libraries, TEX treatment was omitted but 2 µL of 10× Terminator^TM^ Reaction Buffer A and 20 U of SUPERase In RNase Inhibitor were added. All the samples were incubated at 30 °C for 1 h, and the reaction was then terminated by adding 1 µL of 100 mM EDTA (pH 8.0). To ligate the 5′ RNA adaptor, 20 U of RNA 5′-polyphosphatase (Epicentre, Madison, USA) was added to the TEX+ and TEX− samples. For the TAP− sample, the RNA 5′-polyphosphatase treatment was omitted. Two microliters of 10× RNA 5′-phosphatase Reaction buffer (Epicentre, Madison, USA) was added to all samples with 20 U of SUPERase In RNase Inhibitor and incubated at 37 °C for 1 h. The samples were purified by phenol-chloroform extraction and ethanol precipitation. The 5′ RNA adaptor (5′-GUUCAGAGUUCUACAGUCCGACGAUC-3′) (10 pM) was added to the purified mRNA with 2 µL of T4 RNA ligase buffer (Thermo Scientific, Waltham, USA), 2 µL of BSA (1 mg mL^−1^) (Thermo Scientific, Waltham, USA), 20 U T4 RNA ligase (Thermo Scientific, Waltham, USA), and 20 U of SUPERase In RNase Inhibitor (Epicentre, Madison, USA). The samples were then incubated at 37 °C for 90 min, followed by incubation at 70 °C for 10 min. For cDNA synthesis, 10 pM of the random hexamer 3′ overhanging primer (5′-GTGACTGGAGTTCAGACGTGTGCTCTTCCGATCTNNNNNN-3′) was added to the purified sample along with 1 µL of 10 mM dNTPs (Invitrogen, Waltham, USA) and incubated at 65 °C for 10 min. Subsequently, 2 µL of 10× RT buffer, 2 µL of 100 mM DTT, 4 µL of 25 mM MgCl_2_, 20 U of SUPERase In RNase Inhibitor (Epicentre, Madison, USA), and 200 U of SuperScript III Reverse Transcriptase (Invitrogen, Waltham, USA) were added. The reaction was incubated at 25 °C for 10 min, at 50 °C for 60 min, 85 °C for 5 min, and then cooled down to 4 °C. To remove the remaining RNA, 2 U of RNase H (Invitrogen, Waltham, USA) was added and incubated at 37 °C for 20 min. The cDNA fraction at a size range between 120 bp and 400 bp was purified on 2% agarose gel using MinElute Gel Extraction kit (Qiagen, Hilden, Germany). The purified library was amplified with the indexed primer for Illumina sequencing platform and monitored on the CFX96TM Real-Time PCR Detection System (Bio-Rad, Hercules, USA). Amplification was stopped at the beginning of the saturation point. The amplified cDNA was size-selected at a range between 150 bp and 400 bp, which was then sequenced using the 100 bp read recipe on Illumina Hiseq. 2500.

### Data processing

The sequence reads obtained from dRNA-Seq experiments were trimmed to remove the adapter sequences. The dRNA-Seq reads with shorter than 25 bp and random 3′-overhanging (N9) sequences were removed. Using the CLC Genomics Workbench, the trimmed reads were aligned to the assembled *E*. *limosum* ATCC 8486 genome with the following parameters (mismatch cost = 2, deletion cost = 3, insertion cost = 3, length fraction = 0.8, and similarity fraction = 0.8), and the uniquely mapped reads were retained.

### TSS identification

Initially, TSSs identified using TEX- and TAP- reads were obtained as described previously^60^, and then manually inspected using SignalMap^TM^ (Roche NimbleGen, Inc.) for visualization. The obtained TSSs were combined with TEX + reads to determine TSSs using a previously published method^61^. Among the TSSs located from 300 bp upstream to 100 bp downstream of the respective annotated start codon of each ORF, higher TSS score was classified as primary (P) and the others as secondary (S).

### Motif discovery

Motif discovery was achieved using a previously published method^52^. Conserved sequences were determined by MEME software^32^. The 20 bp upstream sequences of each TSS were extracted and the −10 motif was identified using MEME software. Following the extraction, 8 bp upstream and 7 bp downstream sequences of the conserved sequence were obtained and illustrated using Weblogo. Sequences 50 bp and 20 bp upstream of TSS were extracted to obtain the −35 motif, and the 4 bp upstream and 6 bp downstream sequences were then obtained. The obtained sequence was illustrated as mentioned above. The space between the −10 motif and −35 motif was calculated.

### Rapid amplification of 5′ cDNA ends

5′-Tag-cDNA library was constructed as described previously^52^. The RNA samples used for dRNA-Seq library preparation were used to amplify the downstream regions of selected TSSs. The short RNA adapter (5′-ACGGACUAGAAGAAA-3′) (50 µM) was added to 1 µg of the RNA sample with 10 U of T4 RNA ligase (Thermo Scientific), 2 µL of T4 RNA ligase buffer, 2 µL of BSA (1 mg mL^−1^), and 20 U of SUPERase In RNase Inhibitor. The reaction was incubated at 37 °C for 90 min and then at 70 °C for 10 min. After purification, half of the purified sample was treated with 20 U of TAP, which was omitted for the other half as a negative control. The samples were incubated at 37 °C for 1 h. Following purification, 10 µM of RNA adapter (5′-AUAUGCGCGAAUUCCUGUAGAACGAACACUAGAAGAAA-3′), 10 U of T4 RNA ligase (Thermo Scientific), 2 µL of T4 RNA ligase buffer, 2 μL of BSA (1 mg mL^−1^), and 20 U of SUPERase In RNase Inhibitor was added to the samples. The ligated RNAs were purified by ethanol precipitation, and then converted to cDNAs using SuperScript III Reverse Transcriptase. The cDNAs were amplified using 10 µM of cDNA-specific primers and 25 µM of the primer complementary to the latter ligated RNA (5′-GCGCGAATTCCTGTAGAACG-3′). The primer sequences used are listed in Supplementary Table 10.

## Electronic supplementary material

Supplementary Table 3

Supplementary Table 5

Supplementary Table 7

Supplementary Table 8

Supplementary Table 9

Supplementary Information
