# Supplementary material for: Determination of the Genome and Primary Transcriptome of Syngas Fermenting Eubacterium limosum ATCC 8486
Source: Sci Rep. 2017 Oct 20;7:13694. doi: 10.1038/s41598-017-14123-3 (PMC5651825; doi:10.1038/s41598-017-14123-3)
Supplement: Supplementary file 6 — Supplementary Information [file 41598_2017_14123_MOESM6_ESM.doc]

Supplementary Information

**Determination of the Genome and Primary Transcriptome of Syngas Fermenting *Eubacterium limosum* ATCC8486**

Yoseb Song^1^, Jongoh Shin^1^, Yujin Jeong^1^, Sangrak Jin^1^, Jung-Kul Lee^2^, Dong Rip Kim^3^, Sun Chang Kim^1,4,5^, Suhyung Cho^1,4^*, and Byung-Kwan Cho^1,4,5^*

^1^Department of Biological Sciences and KI for the BioCentury, Korea Advanced Institute of Science and Technology, Daejeon 34141, Republic of Korea

^2^Department of Chemical Engineering, Konkuk University, Seoul 05029, Republic of Korea

^3^Department of Mechanical Engineering, Hanyang University, Seoul 133-791, Republic of Korea

^4^KAIST Institute for the BioCentury, Korea Advanced Institute of Science and Technology, Daejeon 34141, Republic of Korea

^5^Intelligent Synthetic Biology Center, Daejeon 34141, Republic of Korea

^*^Correspondence and requests for materials should be addressed to S.C. (email: [shcho95@gmail.com](mailto:shcho95@gmail.com)) and B.-K.C. (email: [bcho@kaist.ac.kr](mailto:bcho@kaist.ac.kr))


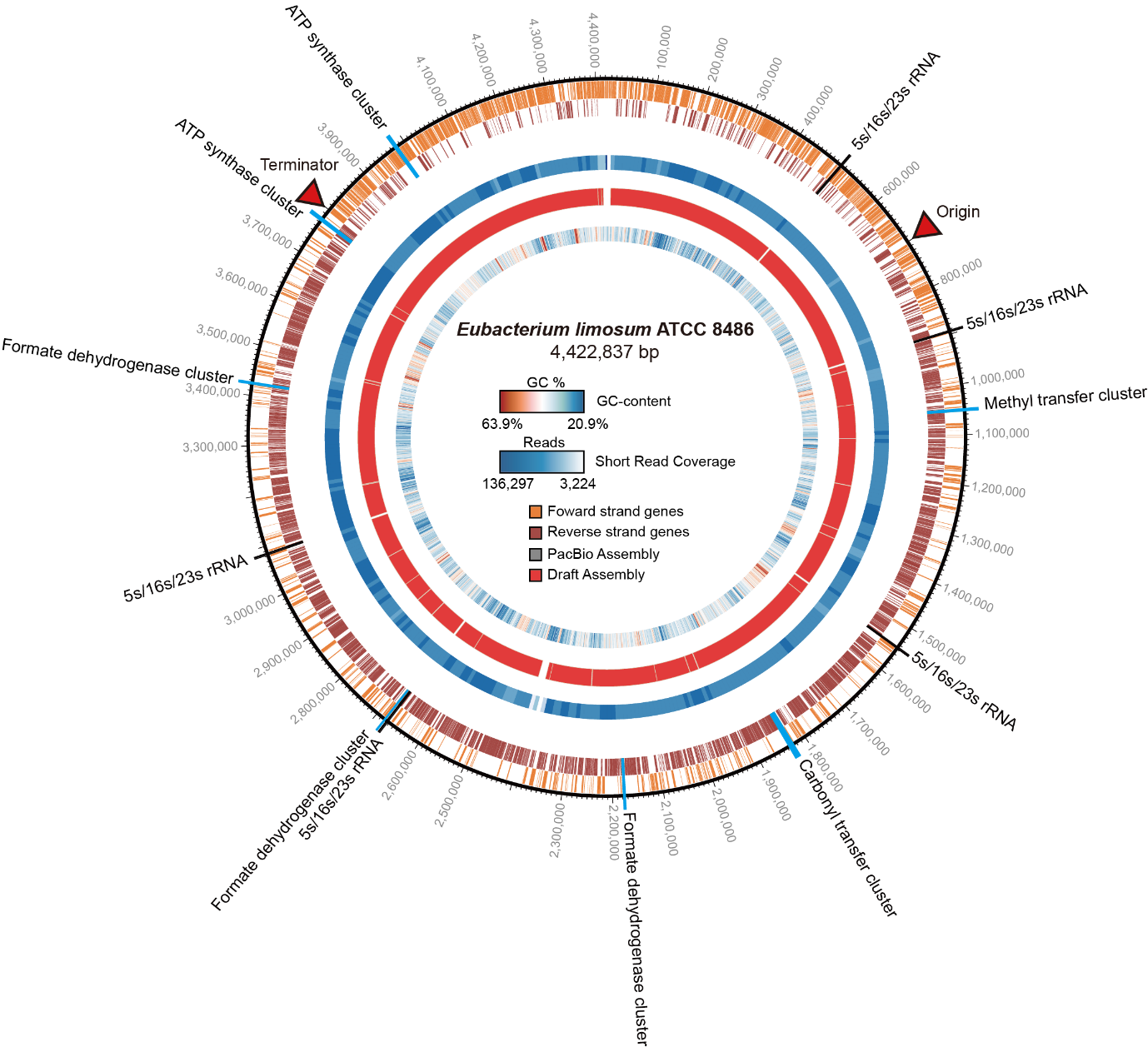


**Supplementary Figure 1 │ Error corrected *Eubacterium limosum* ATCC8486 genome map.** From outside to the centre: Forward strand genes (orange), Reverse strand genes (brown), PacBio assembled contig (grey), Illumina reads coverage (colour by read coverage), the draft genome (red), and GC content (colour by GC%). Red triangles indicate the origin of replicate (genome position of 689,833) and terminus replication (genome position of 3,794,077), respectively. Key genes are labelled with black colour.

**
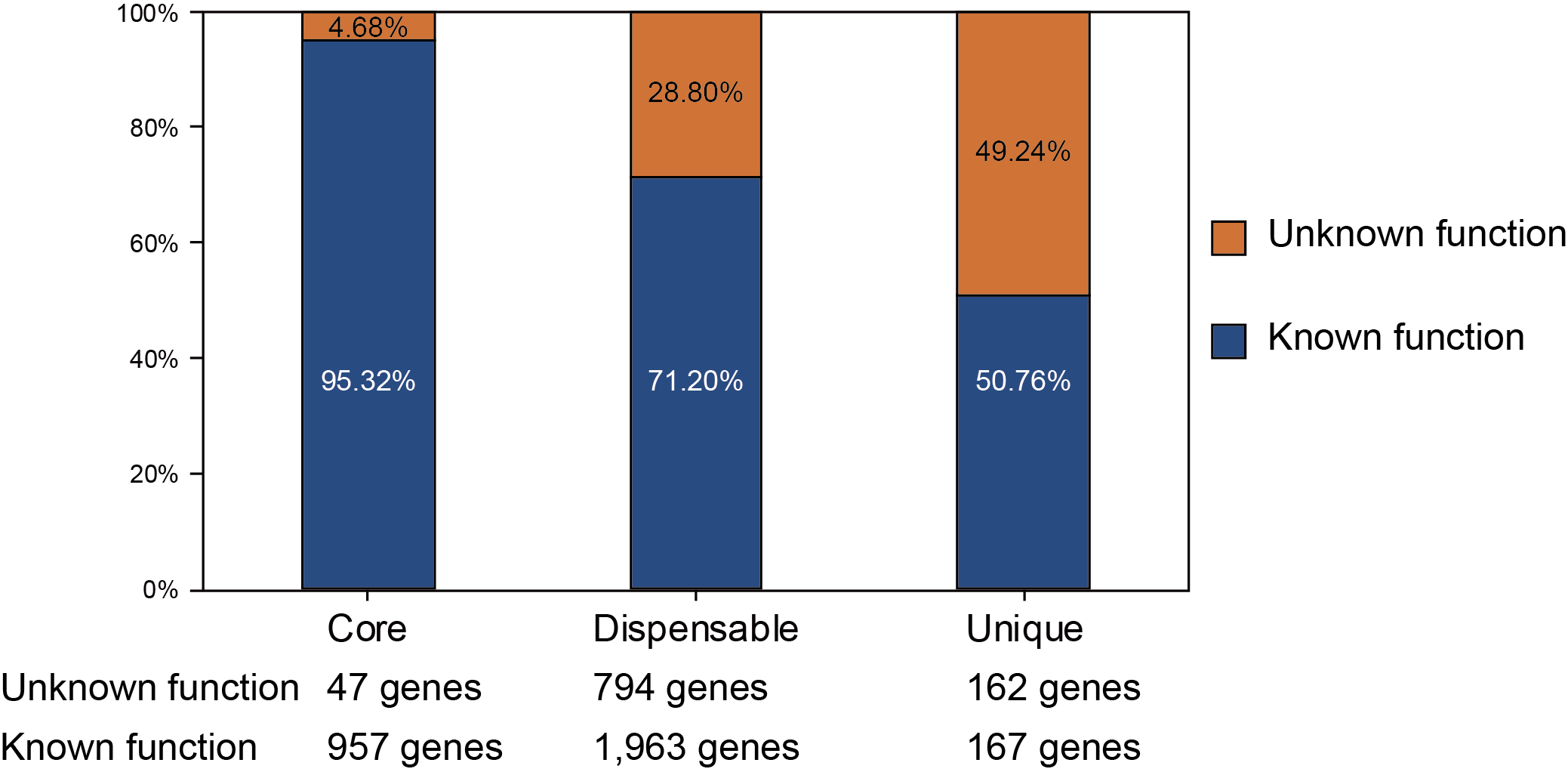
**

**Supplementary Figure 2 │ Pan-genome analysis of *Eubacterium limosum* ATCC8486.** Distribution of known (brown) and unknown protein function (blue) in core, dispensable, and unique gene groups of *E. limosum*.

**
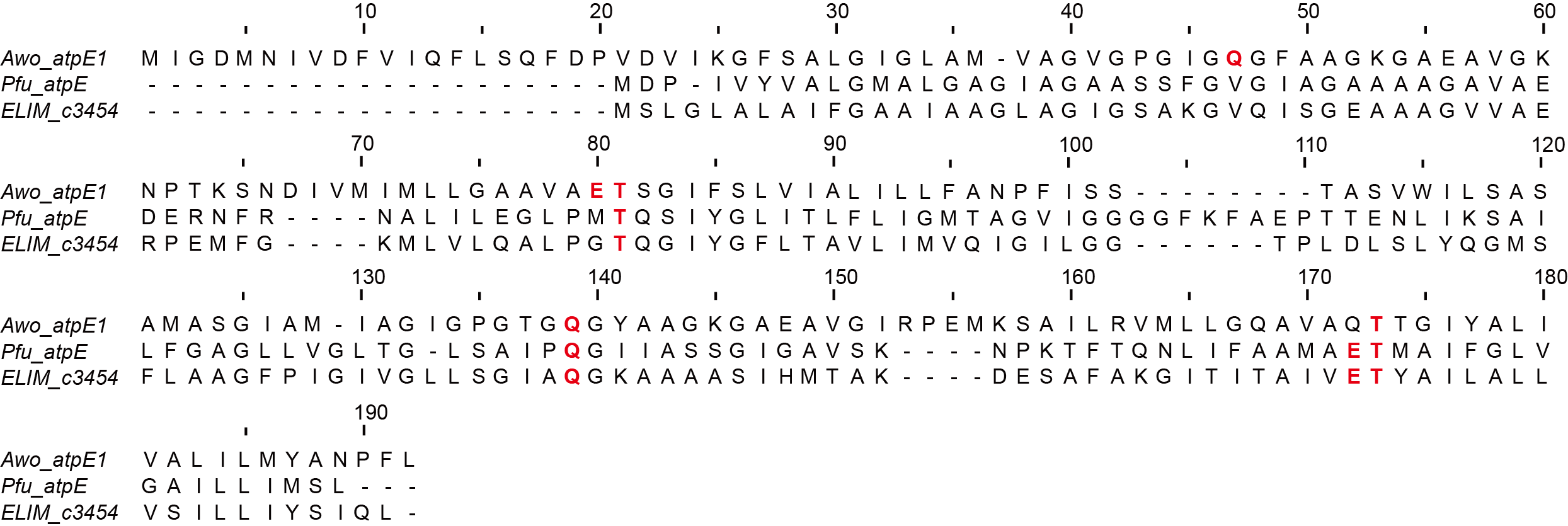
**

**Supplementary Figure 3 │ Alignment of amino acid sequence of the *c* subunit of ATP synthase between *Acetobacterium woodii* (Awo_c02160), *Pyrococcus furiosus* (PF0178), and *E. limosum* ATCC8486 (ELIM_c3454).** The Red bold colour indicates the Na^+^ binding site in ATP synthase.


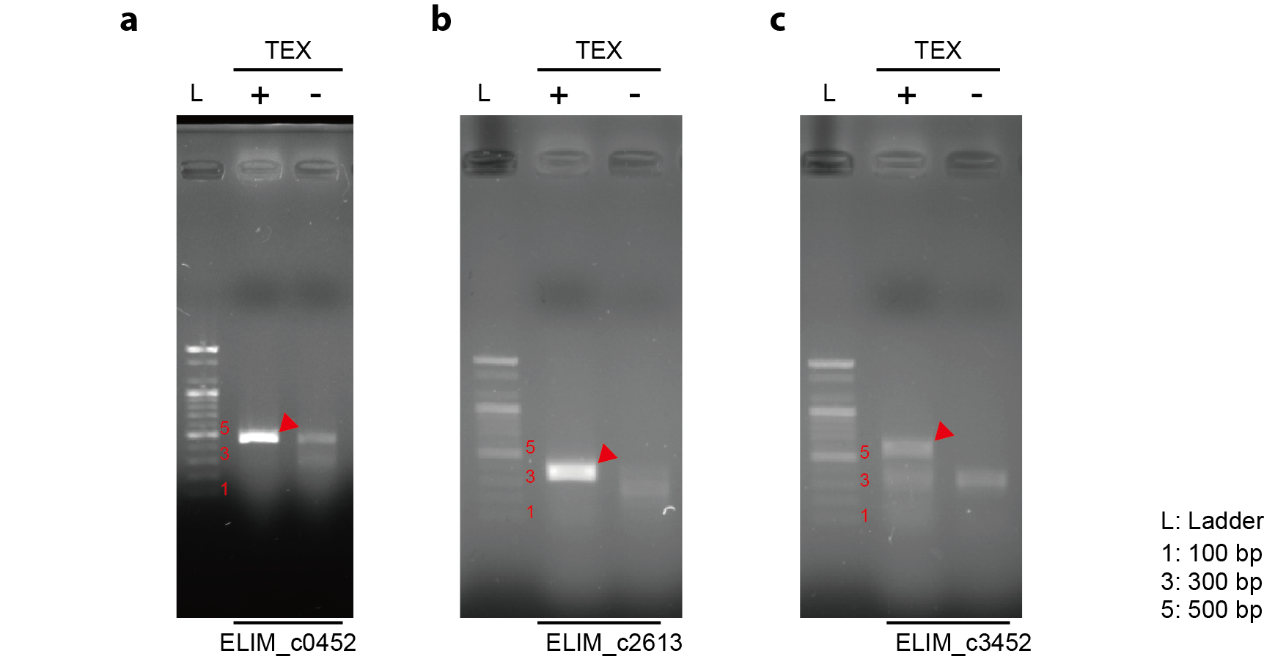


**Supplementary Figure 4 │ Targets of rapid amplification of 5’ cDNA ends.** TSS confirmation of enolase (ELIM_c0452), inosine-5’-monophosphate dehydrogenase (ELIM_c2613), and ATP synthesis subunit (ELIM_c3452) associated genes. The red triangles indicate the target sizes of the amplicons and the red numbers indicate the lengths of markers.


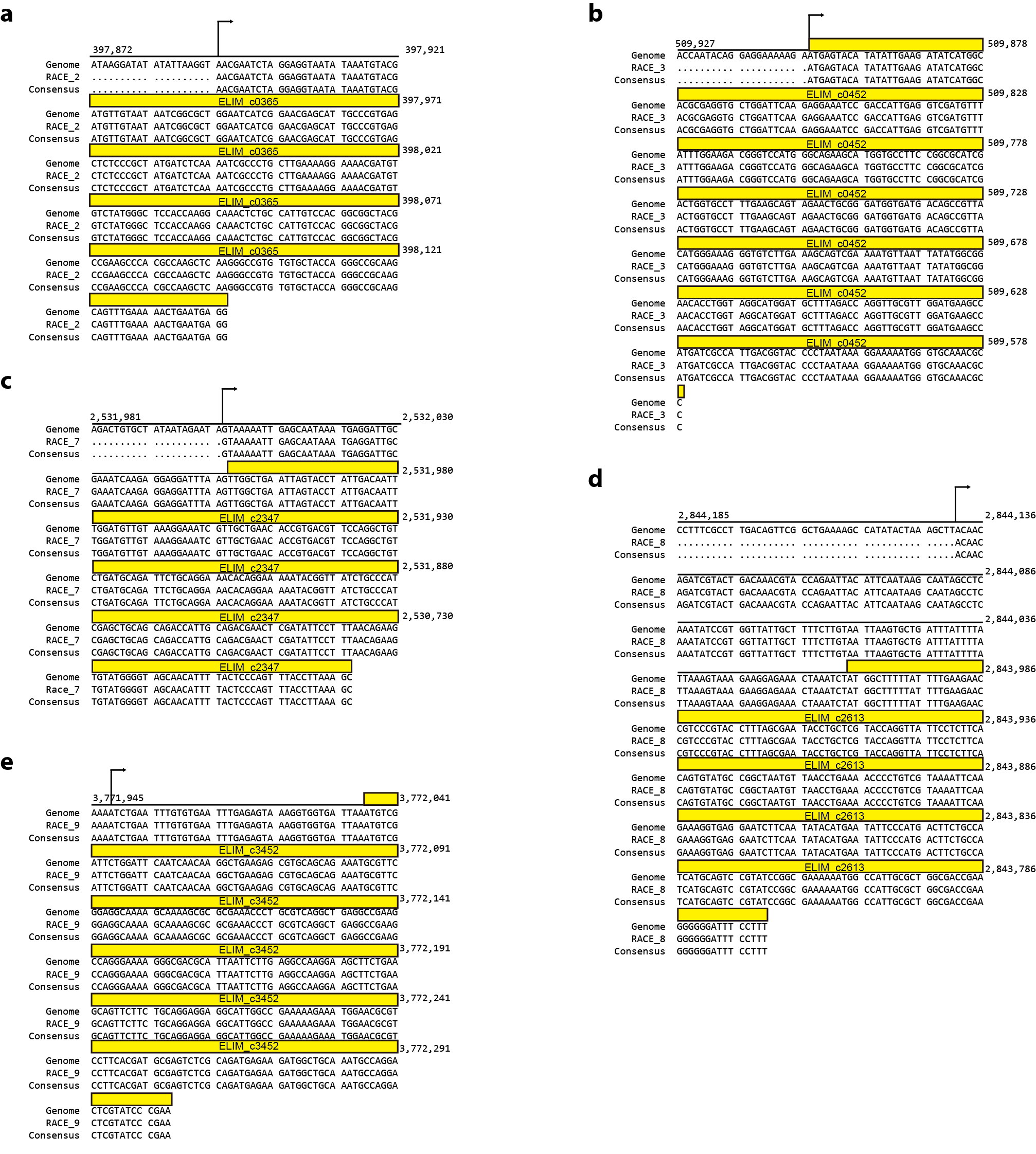


**Supplementary Figure 5 │ Transcriptional start site validation using Sanger sequencing.** Genes encoding glycerol-3-phosphate dehydrogenase (**a**), enolase (**b**), NAD-reducing dehydrogenase (**c**), inosine-5’-monophate dehydrogenase (**d**), and ATP synthase (**e**) TSSs were confirmed using Sanger sequencing. In the left column, the row labelled “Genome” indicates TSSs determined by dRNA-Seq and the row labelled “RACE” indicates amplicon sequence confirmed via Sanger sequencing. The right column describes genomic position. The sequences of primers used are listed in Supplementary Table 9.


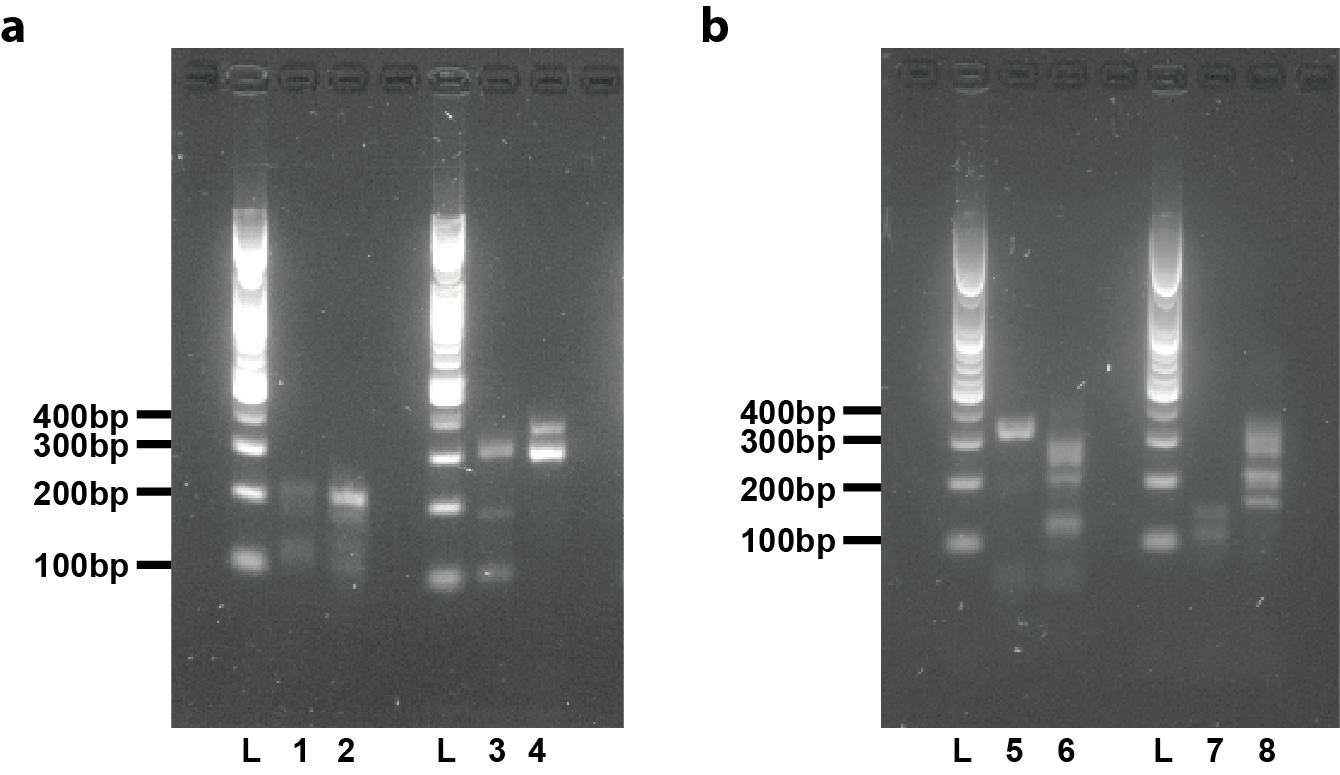


**Supplementary Figure 6 | Full length agarose gel electrophoresis of rapid amplification of 5’ cDNA ends.** Full length of **Figure 3d** that used 2% agarose gel stained by RedSafe. Left panel of Figure 3d (**a**) and right panel of Figure 3d (**b**). Lane 1 and 2: TEX treated and untreated ELIM_c0234 (acetyl-CoA C-acetyltransferase). Lane 3 and 4: TEX treated and untreated ELIM_c0365 (glycerol-3-phosphate dehydrogenase). Lane 5 and 6: TEX treated and untreated ELIM_c2347 (NADH dehydrogenase). Lane 7 and 8: TEX treated and untreated ELIM_c3112 (Rnf2 transcriptional regulation). L represent 100 bp ladder.

**Supplementary Table 1 │** Summary of *E. limosum* genome sequencing statistics

|  |  | Illumina | PacBio RS |
| --- | --- | --- | --- |
| Sequencing | Total Raw Reads | 40,895,842 | 150,292 |
|  | Total Raw Bases (bp) | 6,152,054,301 | 1,138,905,996 |
|  | Average Read Length (bp) | 150.43 | 7,577 |
| Pre-assembly | Number of Reads used in Assembly | 38,752,997 | 8,986 |
|  | Average Read Length (bp) | 150.35 | 4,862 |
|  | Number of Bases used in Assembly (bp) | 5,826,333,852 | 994,287,089 |
| Assembly | Number of Contigs | 31 | 1 |
|  | Maximum Contig Size (nt) | 724,448 | 4,423,093 |
|  | N50 (nt) | 388,816 | 4,423,093 |
|  | Genome size (bp) | 4,370,113 | 4,423,093 |

**Supplementary Table 2 │** Error correction in the assembled genome sequence

|  | Genomic position | PacBio RS | Illumina | RNA-seq | Corrected nucleotide | Types of mutation |
| --- | --- | --- | --- | --- | --- | --- |
| 1 | 806299-806305 | TAC**T**CTT | TAC**C**CTT | TAC**C**CTT | TAC**C**CTT | Mismatch |
| 2 | 1072907-1072912 | GCT*****GGG | GCT**G**GGG | GCT*****GGG | GCT**G**GGG | Deletion |
| 3 | 1971207-1971212 | ACT*****CCC | ACT**C**CCC | ACT**C**CCC | ACT**C**CCC | Deletion |
| 4 | 2386044-2386049 | GGG*****CCC | GGG**C**CCC | GGG**C**CCC | GGG**C**CCC | Deletion |
| 5 | 2386896-2386901 | TTT*****AAA | TTT**A**AAA | TTT**A**AAA | TTT**A**AAA | Deletion |
| 6 | 2387046-2387051 | AAT*****AAA | AAT**A**AAA | AAT**A**AAA | AAT**A**AAA | Deletion |
| 7 | 2393649-2393655 | TGG**T**CTT | TGG**G**CTT | ND | TGG**G**CTT | Mismatch |
| 8 | 3180993-3180996 | AG*******AG | AG**AGC**AG | AG*******AG | AG**AGC**AG | Deletion |
| 9 | 3181140-3181145 | TAT*****CCT | TAT**C**CCT | TAT**C**CCT | TAT**C**CCT | Deletion |
| 10 | 3181236-3181241 | GAT*****GGG | GAT**G**GGG | GAT**G**GGG | GAT**G**GGG | Deletion |
| 11 | 3181420-3181425 | TAT*****GGG | TAT**G**GGG | TAT**G**GGG | TAT**G**GGG | Deletion |
| 12 | 3181445-3181450 | TTT*****CAA | TTT**C**CAA | TTT**C**CAA | TTT**C**CAA | Deletion |
| 13 | 3181454-3181459 | AGA*****GTC | AGA**G**GTC | AGA**G**GTC | AGA**G**GTC | Deletion |
| 14 | 3181576-3181581 | TTT*****CCA | TTT**G**CCA | TTT**G**CCA | TTT**G**CCA | Deletion |
| 15 | 3181584-3181589 | ACT*****ATG | ACT**C**ATG | ACT**C**ATG | ACT**C**ATG | Deletion |
| 16 | 3181588-3181593 | TGT*****CGG | TGT**C**CGG | TGT**C**CGG | TGT**C**CGG | Deletion |
| 17 | 3181594-3181599 | CGA*****CTT | CGA**G**CTT | CGA**G**CTT | CGA**G**CTT | Deletion |
| 18 | 3181601-3181606 | ATG*****TTC | ATG**T**TTC | ATG**T**TTC | ATG**T**TTC | Deletion |
| 19 | 3181614-3181619 | GAC*****TTT | GAC**A**TTT | GAC**A**TTT | GAC**A**TTT | Deletion |
| 20 | 3183274-3183278 | AC*****A*****CA | AC**T**A**T**CA | AC**T**A**T**CA | AC**T**A**T**CA | Deletion |
| 21 | 3183279-3183282 | A******GG*****A | A**TT**GG**T**A | A**TT**GG**T**A | A**TT**GG**T**A | Deletion |
| 22 | 3183287-3183292 | AAA*****GTC | AAA**G**GTC | AAA**G**GTC | AAA**G**GTC | Deletion |
| 23 | 3183292-3183297 | CGA*****TTC | CGA**T**TTC | CGA**T**TTC | CGA**T**TTC | Deletion |
| 24 | 3183300-3183305 | AGA*****ATT | AGA**T**ATT | AGA**T**ATT | AGA**T**ATT | Deletion |
| 25 | 3183312-3183317 | ACC*****GAC | ACC**A**GAC | ACC**A**GAC | ACC**A**GAC | Deletion |
| 26 | 3183323-3183328 | TTT*****GGA | TTT**C**GGA | TTT**C**GGA | TTT**C**GGA | Deletion |
| 27 | 3183327-3183333 | GAA**A**AAG | GAA**G**AAG | GAA**G**AAG | GAA**G**AAG | Mismatch |
| 28 | 3183341-3183345 | CTG*****GC | CTG**A**GC | CTG**A**GC | CTG**A**GC | Deletion |
| 29 | 3183346-3183349 | A******TTA | A**TC**TTA | A**TC**TTA | A**TC**TTA | Deletion |
| 30 | 3183362-3183366 | TC******TAA | TC**TG**TAA | TC**TG**TAA | TC**TG**TAA | Deletion |
| 31 | 3183371-3183374 | T*****AG******T | T**A**AG**GT**T | T**A**AG**GT**T | T**A**AG**GT**T | Deletion |
| 32 | 3183375-3183379 | A*****TTG*****T | A**C**TTG**C**T | A**C**TTG**C**T | A**C**TTG**C**T | Deletion |
| 33 | 3183380-3183384 | CA*****A*****AT | CA**T**A**C**AT | CA**T**A**C**AT | CA**T**A**C**AT | Deletion |
| 34 | 3183391-3183396 | GCA*****ATG | GCA**T**ATG | GCA**T**ATG | GCA**T**ATG | Deletion |
| 35 | 3183403-3183406 | AA**********AA | AA**TTTTTC**AA | AA**TTTTTC**AA | AA**TTTTTC**AA | Deletion |
| 36 | 3183423-3183429 | TAA**A**GAC | TAA**C**GAC | ND | TAA**C**GAC | Mismatch |
| 37 | 3183547-3183553 | C**T***GG**G***AA | C***A**GG***C**AA | C******GG***C**AA | C**GA**GG**GC**AA | Deletion/  Mismatch |
| 38 | 3183566-3183569 | C******A*****AT | C**CT**A**C**AT | C**CT**A**C**AT | C**CT**A**C**AT | Deletion |
| 39 | 3183569-3183573 | TTA*****CA | TTA**G**CA | TTA**G**CA | TTA**G**CA | Deletion |
| 40 | 3183572-3183576 | CA***G**T*****C | CA**CC**T**A**C | CA**CC**T**A**C | CA**CC**T**A**C | Deletion/  Mismatch |
| 41 | 3183584-3183590 | A*****AATGA*****T | A**T**AATGA**T**T | A**T**AATGA**T**T | A**T**AATGA**T**T | Deletion |
| 42 | 3183594-3183599 | TCA*****CAT | TCA**C**CAT | TCA**C**CAT | TCA**C**CAT | Deletion |
| 43 | 3183610-3183614 | T*******GAT*****A | T**TTA**GAT**A**A | T**TTA**GAT**A**A | T**TTA**GAT**A**A | Deletion |
| 44 | 3183623-3183628 | AAG*****TTC | AAG**A**TTC | AAG**A**TTC | AAG**A**TTC | Deletion |
| 45 | 3183633-3183636 | GA*******GT | GA**AAT**GT | GA**AAT**GT | GA**AAT**GT | Deletion |
| 46 | 3183640-3183645 | TCA*****TTC | TCA**T**TTC | TCA**T**TTC | TCA**T**TTC | Deletion |
| 47 | 3183646-3183650 | TG******ATA | TG**AA**ATA | TG**AA**ATA | TG**AA**ATA | Deletion |
| 48 | 3183656-3183660 | A*****TA*****TA | A**T**TA**T**TA | A**T**TA**T**TA | A**T**TA**T**TA | Deletion |
| 49 | 3183664-3183669 | TAG*****CAG | TAG**T**CAG | TAG**T**CAG | TAG**T**CAG | Deletion |
| 50 | 3183682-3183686 | AT*****CA*****T | AT**A**CA**T**T | AT**A**CA**T**T | AT**A**CA**T**T | Deletion |
| 51 | 3183688-3183693 | TTT*****TTT | TTT**G**TTT | TTT**G**TTT | TTT**G**TTT | Deletion |
| 52 | 3183704-3183709 | AT*****A**T**TT | AT**A**A**G**TT | AT**A**A**G**TT | AT**A**A**G**TT | Deletion/  Mismatch |
| 53 | 3183721-3183725 | TA*****T*****AT | TA**G**T**G**AT | TA**G**T**G**AT | TA**G**T**G**AT | Deletion |
| 54 | 3183730-3183733 | A******TG*****C | A**GC**TG**C**C | A**GC**TG**C**C | A**GC**TG**C**C | Deletion |
| 55 | 3183734-3183739 | ATT*****GCG | ATT**C**GCG | ATT**C**GCG | ATT**C**GCG | Deletion |
| 56 | 3183751-3183756 | AAC*****AGC | AAC**T**AGC | AAC**T**AGC | AAC**T**AGC | Deletion |
| 57 | 3183757-3183762 | A*****ATC*****A*****T | A**C**ATC**G**A**G**T | A**C**ATC**G**A**G**T | A**C**ATC**G**A**G**T | Deletion |
| 58 | 3183778-3183781 | T******G*****CC | T**TA**G**A**CC | T**TA**G**A**CC | T**TA**G**A**CC | Deletion |
| 59 | 3183791-3183794 | CA*******GC | CA**AAG**GC | CA*******GC | CA**AAG**GC | Deletion |
| 60 | 3183795-3183799 | T******TCT*****C | T**TA**TCT**C**C | T******TCT*****C | T**TA**TCT**C**C | Deletion |
| 61 | 3183802-3183806 | AT*****T*****TA | AT**A**T**G**TA | AT*****T*****TA | AT**A**T**G**TA | Deletion |
| 62 | 3183809-3183813 | GAC*******AC | GAC**CAA**AC | GAC*******AC | GAC**CAA**AC | Deletion |
| 63 | 3183814-3183819 | C*****TGAG*****T | C**A**TGAG**T**T | C*****TGAG*****T | C**A**TGAG**T**T | Deletion |
| 64 | 3183820-3183824 | AA******ATT | AA**AG**ATT | AA***G**ATT | AA**AG**ATT | Deletion |
| 65 | 3202846-3202851 | TTT*****GGG | TTT**G**GGG | TTT**G**GGG | TTT**G**GGG | Deletion |
| 66 | 3804048-3804054 | TCA**A**CGC | TCA**C**CGC | TCA**C**CGC | TCA**C**CGC | Mismatch |
| 67 | 4419931-4419937 | ATT**T**CCA | ATT**C**CCA | ND | ATT**C**CCA | Mismatch |
| 68 | 4419963-4419969 | TT**T**CC**C**A | TT**C**CC**A**A | ND | TT**C**CC**A**A | Mismatch |
| 69 | 4419975-4419981 | C**T**CCG**C**A | C**C**CCG**A**A | ND | C**C**CCG**A**A | Mismatch |
| 70 | 4419987-4419993 | ATC**T**CCA | ATC**C**CCA | ND | ATC**C**CCA | Mismatch |

**Supplementary Table 4 │ Features of completed genomes of acetogenic bacteria**

| **Organism** | **Size (bp)** | **% GC** | **CDS** | **Coding Density (%)** | **tRNA** | **Opt. Temp** |
| --- | --- | --- | --- | --- | --- | --- |
| *Acetobacterium woodii* DSM 1030^1^ | 4,044,772 | 39.3 | 3,521 | 85.7 | 58 | 30 °C |
| *Acetohalobium arabaticum* DSM 5501^2^ | 2,469,596 | 36.6 | 2,286 | 85.9 | 67 | 30 °C |
| *Carboxydothermus hydrogenoformans* Z-2901^3^ | 2,401,520 | 42.0 | 4,206 | 90.5 | 49 | 78 °C |
| *Clostridium aceticum* DSM 1496^4^ | 4,201,318 | 35.3 | 3,711 | 84.7 | 74 | 30 °C |
| *Clostridium autoethanogenum* DSM 10061^5^ | 4,352,205 | 31.1 | 3,741 | 84.5 | 67 | 37 °C |
| *Clostridium carboxidivorans* P7^6^ | 5,732,880 | 29.9 | 5,004 | 84.2 | 71 | 37 °C |
| *Clostridium ljungdahlii* DSM 13528^7^ | 4,630,065 | 31.1 | 4,081 | 84.2 | 70 | 37 °C |
| *Clostridium scatologenes* ATCC 25775^8^ | 5,749,410 | 29.6 | 4,974 | 85.0 | 63 | 37 °C |
| *Eubacterium limosum* ATCC8486 | 4,422,837 | 47.2 | 4,292 | 88.9 | 60 | 37 °C |
| *Eubacterium limosum* KIST612^9^ | 4,316,707 | 47.5 | 3,966 | 88.4 | 59 | 37 °C |
| *Moorella thermoacetica* ATCC 39073^10^ | 2,628,784 | 55.8 | 2,460 | 86.2 | 51 | 58 °C |
| *Peptoclostridium difficile* 630^11^ | 4,290,252 | 29.1 | 3,756 | 83.5 | 87 | 37 °C |
| *Thermacetogenium phaeum* DSM 12270^12^ | 2,939,057 | 53.9 | 2,766 | 84.0 | 50 | 58 °C |
| *Thermoanaerobacter kivui* DSM 2030^13^ | 2,397,824 | 35.0 | 2,198 | 85.3 | 58 | 60 °C |
| *Treponema primitia* ZAS-2^14^ | 4,059,867 | 50.8 | 3,427 | 89.8 | 51 | 30 °C |

1. Poehlein A, et al. An ancient pathway combining carbon dioxide fixation with the generation and utilization of a sodium ion gradient for ATP synthesis. *PLoS ONE* 7, e33439 (2012).
2. Sikorski J, et al. Complete genome sequence of *Acetohalobium arabaticum* type strain (Z-7288). *Stand Genomic Sci* 3, 57-65 (2010).
3. Wu M, et al. Life in hot carbon monoxide: the complete genome sequence of *Carboxydothermus hydrogenoformans* Z-2901. *PLoS Genet* 1, e65 (2005).
4. Poehlein A, et al. The Complete Genome Sequence of *Clostridium aceticum*: a Missing Link between Rnf- and Cytochrome-Containing Autotrophic Acetogens. *mBio* 6, e01168-01115 (2015).
5. Brown SD, et al. Comparison of single-molecule sequencing and hybrid approaches for finishing the genome of *Clostridium autoethanogenum* and analysis of CRISPR systems in industrial relevant Clostridia. *Biotechnol Biofuels* 7, 40 (2014).
6. Humphreys CM, et al. Whole genome sequence and manual annotation of *Clostridium autoethanogenum*, an industrially relevant bacterium. *BMC Genomics* 16, 1085 (2015).
7. Köpke M, et al. *Clostridium ljungdahlii* represents a microbial production platform based on syngas. *Proc Natl Acad Sci USA* 107, 13087-13092 (2010).
8. Zhu Z, Guo T, Zheng H, Song T, Ouyang P, Xie J. Complete genome sequence of a malodorant-producing acetogen, *Clostridium scatologenes* ATCC 25775(T). *J Biotechnol* 212, 19-20 (2015).
9. Roh H, et al. Complete genome sequence of a carbon monoxide-utilizing acetogen, *Eubacterium limosum* KIST612. *J Bacteriol* 193, 307-308 (2011).
10. Pierce E, et al. The complete genome sequence of *Moorella thermoacetica* (f. *Clostridium thermoaceticum*). *Environ Microbiol* 10, 2550-2573 (2008).
11. Sebaihia M, et al. The multidrug-resistant human pathogen *Clostridium difficile* has a highly mobile, mosaic genome. *Nat Genet* 38, 779-786 (2006).
12. Oehler D, et al. Genome-guided analysis of physiological and morphological traits of the fermentative acetate oxidizer *Thermacetogenium phaeum*. *BMC Genomics* 13, 723 (2012).
13. Hess V, Poehlein A, Weghoff MC, Daniel R, Müller V. A genome-guided analysis of energy conservation in the thermophilic, cytochrome-free acetogenic bacterium *Thermoanaerobacter kivui*. *BMC Genomics* 15, 1139 (2014).
14. Hugerth LW, et al. DegePrime, a program for degenerate primer design for broad-taxonomic-range PCR in microbial ecology studies. *Appl Environ Microbiol* 80, 5116-5123 (2014).

**Supplementary Table 6 │ Statistics summary of dRNA-Seq result of *Eubacterium limosum***

|  | *Eubacterium limosum* | | | |
| --- | --- | --- | --- | --- |
|  | TEX | | TAP | |
|  | Duplicate 1 | Duplicate 2 | Duplicate 1 | Duplicate 2 |
| Number of Reads | 205,951 | 166,028 | 805,738 | 263,031 |
| Number of Bases | 18,540,388 | 14,946,667 | 75,542,788 | 23,341,481 |
| Average Length (bp) | 90.02 | 90.02 | 93.76 | 88.74 |

**Supplementary Table 10 │** Primer sequences used for TSS confirmation via rapid amplification of 5’ cDNA ends (5’-RACE)

| **Gene** | **Function** | **Strand** | **Primer sequence (5' to 3')** |
| --- | --- | --- | --- |
| ELIM_c0365 | Glycerol-3-phosphate dehydrogenase | Forward | ACGAATCTAGGAGGTAATATA |
|  |  | Reverse | GCGCAAAGCCAAAATTCAGCT |
| ELIM_c0452 | Enolase | Forward | TATCTTCAATATATGTACTCAT |
|  |  | Reverse | AGCCATGGAGATTCCTAAAAT |
| ELIM_c2347 | NADH dehydrogenase | Forward | TAAAAATTGAGCAATAAATGA |
|  |  | Reverse | GCTTTAAGGTAAACTGGGAGT |
| ELIM_c2613 | Inosine-5'-monophosphate dehydrogenase | Forward | TTGTCAGTACGATCTGTTGTA |
|  |  | Reverse | AAAGGAAATCCCCCCTTCGGT |
| ELIM_c3452 | V-ATPase subunit | Forward | TGGGTTTTCTTTTAAATTTTT |
|  |  | Reverse | GCCTTCGGGATACGAGTCCTG |
